# Evaluation of two local drug delivery systems as adjuncts to mechanotherapy as compared to mechanotherapy alone in management of chronic periodontitis: A clinical, microbiological, and molecular study

**DOI:** 10.4103/0972-124X.60224

**Published:** 2009

**Authors:** Sangeeta Singh, Subrata Roy, S. K. Chumber

**Affiliations:** *Graded Specialist, 320 Field Hospital C/0 99 APO, India*; 1 *Officer Commanding, Military Dental Centre, Barrackpore, WB, India*; 2 *Classified Specialist (Microbiology), Base Hospital, Tezpur, Assam, India*

**Keywords:** Local drug delivery, multiplex PCR, periodontal pathogens

## Abstract

**Background:**

Modern concepts of treating inflammatory periodontal disease aim at changes in the subgingival ecosystems within the periodontal pockets to alter the complex microbial community into a microbiota compatible with good periodontal health. Systemic antimicrobial therapy, although effective, involves a relatively high dose with repeated intakes over a prolonged period of time to achieve the required inhibitory concentrations in the sulcular fluid. The adjunctive use of local drug delivery may provide a beneficial response, especially in specific areas where conventional forms of therapy might fail. The aim of this study was to compare the efficacy of two local drug delivery systems, one containing metronidazole and the other containing tetracycline hydrochloride as adjuncts to mechanotherapy in the treatment of chronic periodontitis.

**Materials and Methods:**

There were three groups that were labeled as group A (Scaling + Tetracycline), group B (Scaling + Metronidazole), and group C (Scaling alone). A microbiological analysis was carried out to determine the efficacy of these systems in changing the pathogenic flora in deep pockets. In addition, a multiplex polymerase chain reaction was carried out to confirm the presence of *Actinobacillus actinomycetemcomitans, Porphyromonas gingivalis* (Pg), and *Tannerella forsythensis* in the flora associated with chronic periodontitis.

**Results:**

There was clinical improvement in groups A and B, which correlated with an improvement in the microbiological parameters; these results were sustained for 90 days following therapy. In Group C, the flora showed a shift towards baseline at the end of 90 days.

**Conclusions:**

According to this study, both the local antibiotic therapies resulted in greater improvement in microbiological parameters when used as an adjunct to mechanotherapy as compared to mechanotherapy alone.

## INTRODUCTION

Bacterial etiology of periodontal disease has been explored for over 100 years, with continuous advances in molecular techniques to identify newer species of organisms that may serve as markers of ongoing disease or predictors of future disease.[1] Traditionally, the therapy for periodontitis consisted of scaling and root planing. However, mechanical therapy may, at times, fail to eliminate the pathogenic bacteria in inaccessible areas, leading to recurrence of the disease. Increasing knowledge of anaerobic bacteria as predominant agents in the development of periodontal diseases, has led to new treatment modalities based on systemic or local antimicrobial therapy.

Systemic antimicrobial therapy, although effective, involves a relatively high dose with repeated intakes over a prolonged period of time to achieve the required inhibitory concentrations in the sulcular fluid. This increases the chances of development of resistance, of alterations of the commensal flora, and of increased potential for adverse effects.

Advances in the technology of drug delivery systems have resulted in a number of site-specific, controled release methods. Local delivery systems offer the advantages of high concentrations at the target site with reduced dosage, fewer applications, and high patient acceptability.[2] Thus, adjunctive use of local drug delivery may provide a beneficial response, especially in specific areas where conventional forms of therapy might fail. The local drug delivery systems are especially indicated for patients in maintenance phase, medically compromised patients who cannot undergo surgical therapy, institutionalized patients, localized refractory sites, and failing implants. They are also indicated prior to regenerative surgery to improve the predictability by reducing the bacterial load.[3] We have carried out a study to compare two local drug delivery systems, one containing tetracycline and the other containing metronidazole, as adjuncts to mechanotherapy, and have compared the results using the two antimicrobials with those obtained with mechanotherapy alone.

## MATERIALS AND METHODS

In the present study, 120 subjects were selected from among those patients who had come for treatment in the OPD of the Department of Dental Surgery, Armed Forces Medical College, Pune. All the selected subjects were ascertained to be in good general health with no history of any systemic disease and no history of antibiotic therapy, oral prophylaxis, or periodontal surgery during the last six months. Pregnant or lactating females as well as smokers were excluded. There were 120 patients in the age group of 30-55 years who had at least one site with a probing depth of 5 mm in a minimum of two quadrants.

The study was a single-blind, randomized, parallel group clinical study that was started in February 2005. Clinical measurements and microbiological samples were taken from the selected sites prior to treatment, *i.e*., on day 0, and on days 30 and 90 following treatment. After selection of the cases, the following clinical parameters were assessed: Probing depth (PD), Clinical attachment level (CAL), Plaque index (PI), Gingival index (GI), and Gingival bleeding index (GBI).

### Microbiological analysis

Subgingival plaque samples were collected with a sterile Gracey curette from the (preselected) deepest site in each quadrant. These samples were collected from the sites prior to treatment, and after 30 and 90 days following treatment. The supragingival plaque was removed with a sterile curette and cotton gauze. Subsequently, a subgingival plaque sample was obtained using another sterile Gracey curette. The samples were immediately processed for gram staining and aerobic and anaerobic culture. The media used for anaerobic culture were Dentaid,[4] *N* -acetyl muramic acid (NAM), and Trypticase soy agar (TSBY). After inoculation with the samples, the three media (Dentaid, NAM, and TSBY) were placed in a McIntosh Fildes jar along with heat-activated palladium pellets. The heat-activated palladium combines with any remnant oxygen in the jar to remove it. A tube with citrate medium was inoculated with *Pseudomonas*, an obligate aerobe, and placed along with the culture plates in the jar. The growth of *Pseudomonas* seen on opening the jar after ten days, would indicate the presence of oxygen and hence, serves the purpose of a bacterial indicator to rule out false negative results. The jars were closed and a vacuum was created inside, after which 10% hydrogen gas and 5% carbon dioxide were passed through the inlet of the jar to create an anaerobic environment. The jars were closed tightly and placed in the incubator room where a temperature of 37°C was constantly maintained.

For aerobic culture, the samples were inoculated in blood agar plates and kept at 37°C for 24 hours.

#### Bacterial identification

**Figure 1:**
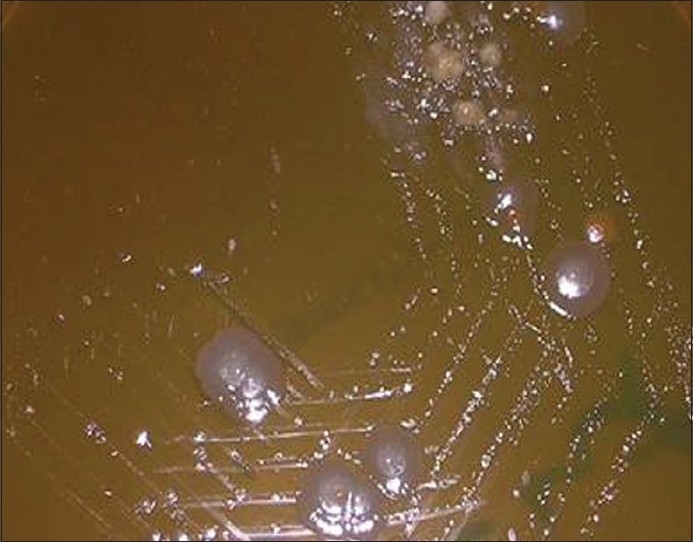
Anaerobic colonies on dentaid medium

The culture plates were checked for any growth after 24 h and the colonies were identified further on the basis of the presence and type of hemolysis and gram staining. Any positive growth seen after opening the culture plates after ten days was checked for colony morphology, color, aerotolerance, and biochemical tests. The colonies were observed for size, shape, borders, color, surface, and changes produced in the medium [Figure 1].

#### Biochemical tests

The colonies were further subjected to a series of biochemical tests for identification: catalase, oxidase, and indole tests.

#### Bacterial counting

For evaluating the change in flora following treatment and to statistically analyze the quantitative change, bacterial counting was done for gram-positive cocci and gram-negative bacilli prior to treatment, and on days 30 and 90 following treatment. Twenty-five fields were counted for each slide and the counts totaled to get the approximate numbers.

### Multiplex polymerase chain reaction

A multiplex PCR reaction was standardized to confirm the presence of *Aggregatibacter actinomycetemcomitans* (Aa), *Tannerella forsythensis* (Tf), *and Porphyromonas gingivalis* (Pg) in the subgingival samples from patients diagnosed with chronic periodontitis. This molecular analysis was carried out to detect the presence of Aa, Pg and Tf in the baseline subgingival plaque samples of patients with periodontitis and to detect their presence 90 days after therapy.

#### Preparation of bacterial templates for DNA extraction

Plaque samples were collected by using sterile paper points which were placed in the pocket for a few seconds and then placed in 70 μL of AE buffer (Commercial kit). These samples were kept at −80°C until DNA extraction could be performed.

#### DNA extraction

This procedure was carried out as per the protocol given by the manufacturer. The DNA templates were stored at −20°C until further use.

#### Primers: Forward (Fwd)

5'-ATT GGG GTT TAG CCC TGG TG-(Aa), 5'-TAC AGG GGA ATA AAA TGA GAT ACG-3' (Tf), 5'-TGT AGA TGA CTG ATG GTG AAA ACC-3' (Pg), and 5'-ACG TCA TCC CCA CCT TCC TC-3' (Conserved reverse primer).

#### Putting up a multiplex PCR

The reaction was set up inside a biosafety cabinet; the volumes of reagents for carrying out a 25 μL reaction are:

- 2.5X PCR premix 10 μL
- Millipore water 3.5 μL
- Fwd primer 1 (Aa) 1.5 μL
- Fwd primer 2 (Pg) 1.5 μL
- Fwd primer 3 (Tf) 1.5 μL
- Rev primer (C11R) 2 μL
- Sample 5 μL

### Total volume 25 μL

We standardized the following temperatures for our multiplex PCR and the thermocycler was programmed accordingly: (i) 95°C for 10 min, (ii) 95°C for 20 sec (Denaturation), (iii) 62°C for 30 sec (Annealing), (iv) 72°C for 30 sec (Extension), (v) 72°C for 5 min. Steps (ii) to (iv) were repeated 40 times and the results were visualized using the gel electrophoresis technique described earlier. Two local drug delivery systems containing the following two components were used: i) 5% metronidazole with 95% collagen in the form of a sponge, and ii) 2 mg tetracycline with 25 mg collagen in the form of fibers. After collection of the baseline data, a thorough subgingival scaling was carried out for all the subjects and the subjects were then randomly divided into three groups designated as A, B, and C. Each group had 40 patients with equal numbers of males and females. The subjects in *Group A* received a single application of tetracycline fibers subgingivally, the subjects in *Group B* received a single application of metronidazole, and *Group C* served as the control group and received no further treatment after subgingival scaling. The patients were not given any instructions in oral hygiene measures. The use of any antibiotics or mouthwash was not permitted during the study period so as to not affect the outcome of therapy. The data obtained were subjected to statistical analysis using various tests to compare the baseline clinical and microbiological parameters of the three groups. The three months' and baseline differences were analyzed statistically using one-way analysis of variance (ANOVA) and in-between groups were compared.

## RESULTS

A total of 120 chronic periodontitis patients between the ages of 30 and 55 years were randomly divided into three groups: A, B and C. Each group comprised of 40 subjects at the start of the study. However, 12 patients did not return for follow-up after treatment. Thus, there were 36 subjects in group A, 37 subjects in group B, and 35 subjects in group C, who completed the 90 days follow-up **[Tables 1, 3 and 5]**. We also standardized a multiplex PCR to detect the presence of Aa, Pg, and Tf at baseline for 45 samples, which was repeated at the 90-days' evaluation. This multiplex PCR was set up to detect the presence of Aa, Pg, and Tf in the baseline subgingival plaque samples of patients with periodontitis and to detect their presence after therapy. The comparative changes in clinical parameters in the three groups are shown in Table 1 and the changes in microbiological parameters in the three groups are shown in Table 2. In *Group A,* there was a predominance of gram-negative bacilli (GNB) and a few gram-negative coccobacilli at baseline. Apart from these, gram-positive cocci (GPC) formed the second predominant group with a few gram-negative bacilli. After 30 days, the smear showed a reduction in the gram-negative organisms and there was a relative predominance of the gram-positive group. This picture further improved after 90 days following treatment and there was a predominantly gram-positive flora and very few gram-negative organisms [Figure 2a–c]. At 30 and 90 days following therapy, a similar shift in flora was observed in *Group B.* The microscopic picture at baseline showed a predominantly gram-negative flora and a lower density of gram-positive organisms. This flora started showing improvement at the 30-days' evaluation and showed a relative decrease in the gram-negative group with an associated increase in gram-positive organisms. After 90 days, there was a marked increase in gram-positive organisms with a reduction in the gram-negative group [Figure 3a–c]. In *Group C*, although the improvement from baseline to 30 days showed a microscopic picture comparable to that of Groups A and B, the flora showed a reversal towards baseline at the 90-days' sample evaluation. There was an increase in the gram-negative group with an associated decrease in gram-positive organisms 90 days following treatment in this group, in which no antibiotic was delivered after subgingival debridement [Figure 4a–c]. Statistical analysis showed that there was a significant difference in the changes in GNB and GPC scores between the three groups [Table 3]. Bonferroni analysis for multiple comparisons of changes in GNB and GPC in the three groups showed that both the treatment groups A and B showed significant improvement as compared to the control group C, and group A and group B treatments were equally effective. In the present study, we had set up multiplex PCR using primers for Aa, Pg, and Tf. The expected product lengths were 360 bp for Aa, 745 bp for Tf, and 197 bp for Pg. The reaction was put up for 15 samples each from the three groups (A, B and C) at baseline and at 90 days after treatment. The results of the PCR showed the presence of Aa, Pg, and Tf in 0/15, 8/15, and 9/15 samples respectively in group A. Of these, Aa was not seen either before or after treatment; Pg was present in 1/15 samples taken posttherapy, whereas Tf was present in 3/15 samples seen posttreatment in group A. In group B, Aa, Pg, and Tf were seen in 0/15, 6/15 and 7/15 of the baseline samples respectively. The posttreatment samples showed Pg and Tf in 2/15 and 1/15 of the samples respectively. Aa was not seen in group C, but Pg and Tf were present in 8/15 and 5/15 of the samples. After therapy, Pg was seen in 5/15 of the samples, whereas Tf was present in 6/15 of the samples [Figure 5].

**Table 1:** Comparison of changes in clinical parameters in the groups A and B following treatment

Group	Probing depth					
	
	Baseline mean (mm)	SD	30 days mean (mm)	SD	90 days mean (mm)	SD
A	6.918	1.137	3.864	0.635	2.364	0.388
B	6.814	1.151	3.857	0.651	2.457	0.415

**Group**	**Clinical attachment loss**					
	
	**Baseline mean (mm)**	**SD**	**30 days mean (mm)**	**SD**	**90 days mean (mm)**	**SD**

A	5.716	0.939	2.581	0.424	1.648	0.27
B	5.4	0.921	2.885	0.487	1.685	0.284

**Group**	**Gingival index**					
	
	**Baseline mean (mm)**	**SD**	**30 days mean (mm)**	**SD**	**90 days mean (mm)**	**SD**

A	2.054	0.337	1.351	0.222	0.972	0.159
B	2.028	0.342	1.371	0.231	1	0.169

**Group**	**Plaque index**					
	
	**Baseline mean (mm)**	**SD**	**30 days mean (mm)**	**SD**	**90 days mean (mm)**	**SD**

A	2.61	0.494	1.72	0.454	1.08	0.280
B	2.54	0.505	1.71	0.426	1.03	0.169

**Group**	**Gingival bleeding index**					
	
	**Baseline mean (mm)**	**SD**	**30 days mean (mm)**	**SD**	**90 days mean (mm)**	**SD**

A	2.54	0.417	1.513	0.248	1	0.164
B	2.628	0.444	1.714	0.289	1.142	0.193

**Table 2:** Comparison of changes in microbiological parameters in group A and B following treatment

Group	Changes in mean count of gram positive cocci following treatment in groups A and B					
	
	Baseline mean (mm)	SD	30 days mean (mm)	SD	90 days mean (mm)	SD
A	1589.324	325.016	2148.513	353.213	2301.675	378.393
B	1558.057	263.359	2166.085	366.135	1930.685	326.345

**Group**	**Changes in mean count of gram negative bacilli following treatment in groups A and B**					
	
	**Baseline mean (mm)**	**SD**	**30 days mean (mm)**	**SD**	**90 days mean (mm)**	**SD**

A	1977	261.283	667.81	109.787	283.027	46.52
B	1558.057	263.359	2166.085	366.135	1930.685	326.345

**Group**	**Percentage increase in gram positive cocci following treatment in groups A and B group from day 0 to day 30 from day 0 to day 90**					
	
	**30 days**				**90 days**	

A	35.18				44.82	
B	39.02				23.91	

**Group**	**Percentage decrease in gram negative bacilli after therapy in groups A and B group from day 0 to day 30 from day 0 to day 90**					
	
	**30 days**				**90 days**	

A	66.22				85.68	
B	69.09				60.39	

**Figure 2a:**
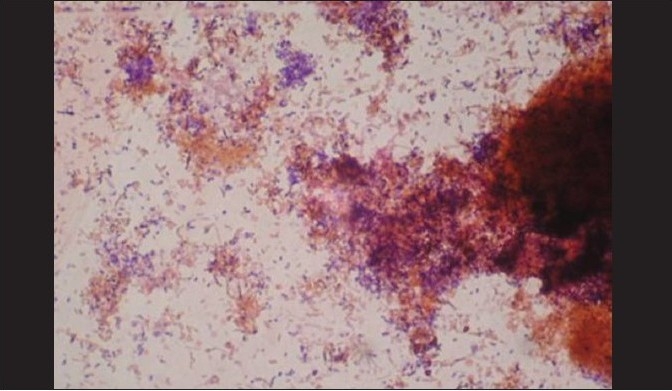
Gram stain with predominance of gram-negative coccobacilli

**Figure 2b:**
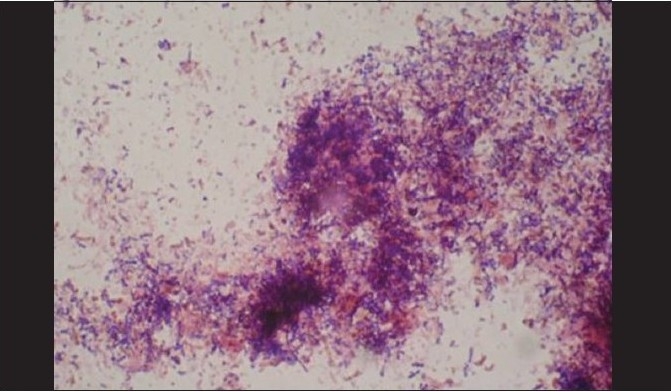
After 30 days, group A smear showed a reduction in the gram-negative organisms with relative predominance of gram-positive group

**Figure 2c:**
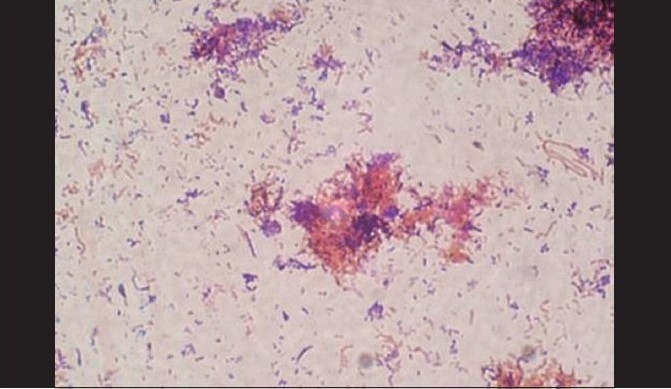
After 90 days, a predominantly gram-positive flora and very few gram- negative organisms (group A)

**Figure 3a:**
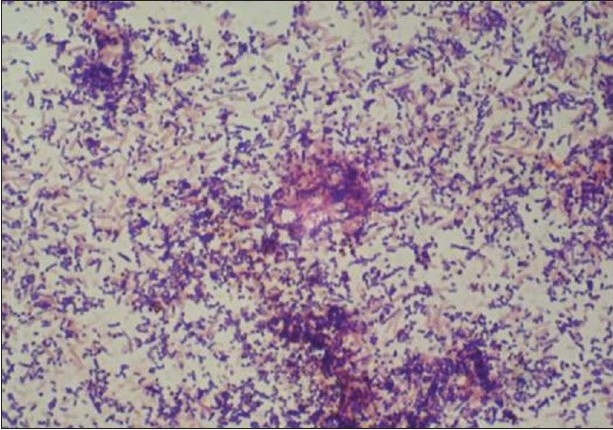
A predominantly gram-negative flora and lesser density of gram-positive organisms at baseline (group B)

**Figure 3b:**
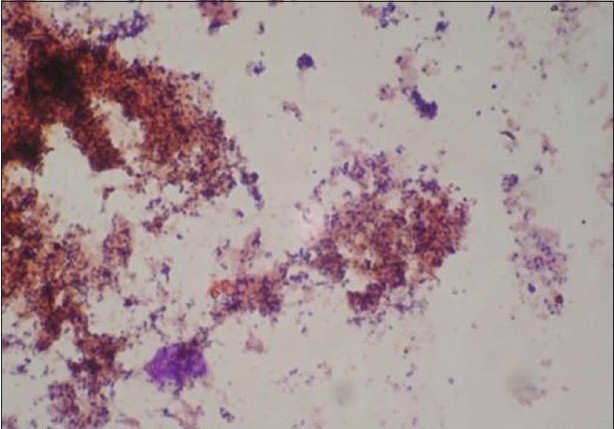
At 30 days evaluation, group B showed a relative decrease in the gram-negative group with associated increase in gram-positive organisms

**Figure 3c:**
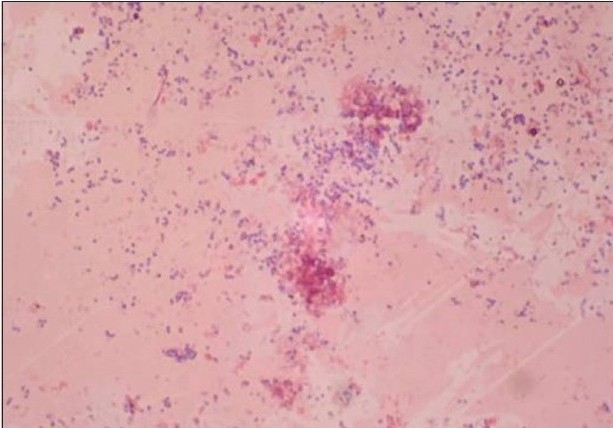
After 90 days, there was a marked increase in gram-positive organisms with reduction in the gram-negative group (group B)

**Figure 4:**
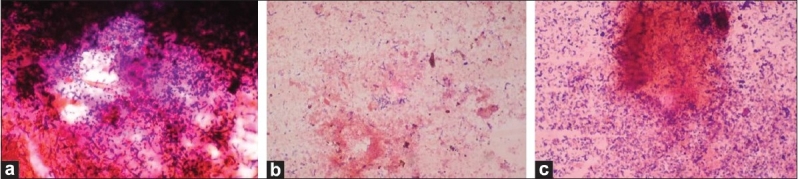
(a) Baseline smear (group C); (b, c) After 30 days, group C showed a microscopic picture comparable to that of groups A and B

**Figure 5:**
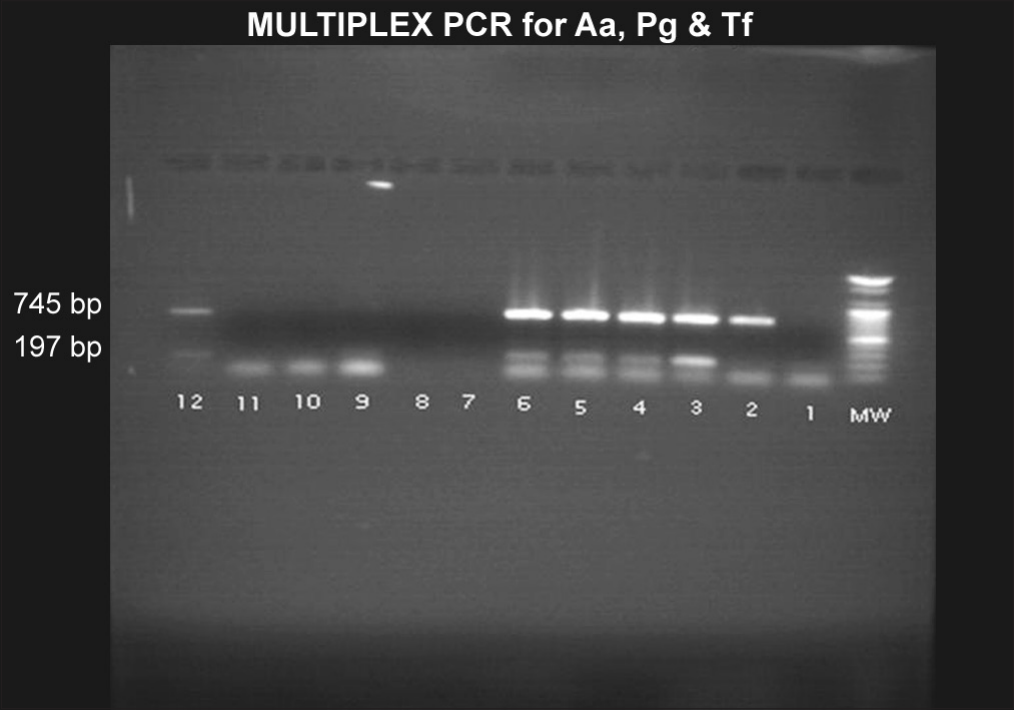
Multiples PCR where Pg and Tf bands are seen

## DISCUSSION

Advances in understanding the etiology and pathogenesis have led to the development and subsequent acceptance of the use of pharmacological agents in the management of periodontal diseases. Local drug delivery systems have the ability to deliver the antibiotic to the target sites, achieve a sufficient concentration, and last for a sufficient duration to be effective. We carried out this study to compare two local drug delivery systems, one containing tetracycline (2 mg) incorporated in collagen fibers (25 mg) and the other containing metronidazole (5%) in a collagen sponge (95%). These systems were used as adjuncts to mechanotherapy in the treatment of chronic periodontitis and the results obtained were compared with those obtained using mechanotherapy alone. The clinical findings were evaluated at baseline, and at 30 and 90 days after therapy. In order to evaluate the efficacy of the treatments in suppressing the pathogenic flora, a microbiological analysis of the subgingival samples was carried out along with the clinical evaluation. The present study evaluated both clinical and microbiological parameters at baseline as well as after the placement of tetracycline and metronidazole in the pockets, and thus, permitted correlation of the results. One of the important criteria given by Socransky for a specific bacterium to be considered as a pathogen, is that it must be present in high numbers at diseased sites and absent in healthy sites.[1] A very limited number of specific periodontal pathogens have been identified using this criterion, including Aa, Pg, and Tf.[5] In earlier studies, cultural data were used to demonstrate the presence of these specific bacteria.[6] However, multiplex PCR has the ability to amplify a single copy of DNA template by several million fold, and by combining multiple primers in a single reaction mixture, it has the ability to identify more than one periodontal pathogen in a single patient sample.[7,8] This extreme sensitivity could be particularly useful in detecting the periodontal pathogens in subgingival plaque samples as their detection in low numbers will identify levels below those detectable by conventional methods.[9] An important aspect of this study was to detect the presence of these periodontal pathogens (Aa, Pg, and Tf) in the subgingival flora at baseline as well as 90 days after therapy, using multiplex PCR.

The earlier studies with tetracycline were carried out using hollow cellulose acetate fibers,[10,11] ethyl vinyl acetate fibers (EVA), and acrylic strips.[12,13] The disadvantage with these systems was that they were nonresorbable and had to be removed. In this study, we have used resorbable collagen fibers loaded with 2 mg tetracycline. Addy *et al.* evaluated the use of local antimicrobial therapy with metronidazole in acrylic strips[14] by using metronidazole as a monotherapy in the treatment of chronic periodontitis and compared it with conventional root instrumentation. They found that metronidazole alone was as effective as root planing until a period of 14 weeks following therapy. However, the disadvantage of the nonresorbable acrylic strips was that they had to be replaced after one week and then subsequently removed In the present study, we have used a collagen sponge impregnated with 5% metronidazole which requires a single placement. This device has been used in two earlier reported studies where a significant improvement in clinical and microbiological parameters was reported following a single application.[15,16] Similar studies comparing scaling and root planing to metronidazole used as an adjunct to scaling and root planing, have shown no significant difference in the results achieved in terms of a reduction of probing depth.[17,18] However, a few studies have reported a significant difference in the improvement in clinical parameters when metronidazole was combined with mechanotherapy, as compared to mechanotherapy alone. In the present study, we found a slightly lower reduction in probing depth in the scaling-only group (C), but this difference was statistically insignificant. The results of this study are in agreement with those of earlier studies where tetracycline was used as an adjunct to mechanotherapy and compared to mechanotherapy alone.[19,20] There was no significant difference reported in the clinical parameters where CAL was assessed. Although there was a slightly lower gain in attachment levels in the scaling group as compared to the tetracycline plus scaling group, the difference was found to be statistically insignificant. However, Minabe *et al.* evaluated tetracycline-impregnated collagen films as an adjunct to root planing in treatment of Grade II furcation defects, and compared the results to root planing alone. They found that the combination therapy enhanced the effects of root planing.[21]

In Group A, there was a change in the microbiological parameters and this consisted of a reduction in GNB count by approximately 85% with an associated increase in GPC counts by 65%. The number of anaerobic colonies also decreased after therapy. These results are comparable to those of previous studies where the effect of a tetracycline-containing local drug delivery system was assessed on subgingival flora in periodontitis.[22] The results observed in Group B were comparable to those seen in Group A and there was a reduction of 65.8% with an associated gain in clinical attachment of 71%. The GNB count reduced by 85.6% and GPC scores increased by 44%. In this group also, there was a reduction in the number of anaerobic colonies. These findings are comparable to those of earlier studies conducted on the effects of a metronidazole-containing local drug delivery system on subgingival flora.[23] The increase in GPC scores and reduction in GNB scores denote a shift in subgingival flora from a pathogenic type to a flora which is compatible with health. An earlier study on microbiologic monitoring of subgingival flora during periodontal therapy, also reported a similar shift after studying motile rods and coccoid cells.[24]

In Group C, the microbiological picture was very different compared to the other two groups, from baseline to 30 days posttreatment. Ninety days after therapy, the reduction in GNB scores was significantly lower than the reduction seen in Groups A and B. Sbordone *et al.* reported similar findings where recolonization of pathogenic flora started 4-8 weeks after scaling in deep pockets.[25] The present study showed a similar pattern where despite the initial improvement and shift towards a healthier flora 30 days after therapy, the reversal of the flora to baseline was noted 90 days after treatment. This microbiological picture 90 days after scaling did not correlate positively with the clinical parameters which showed an improvement from the baseline. A similar finding was reported by Addy *et al*.[14] who suggest that the inflammatory response lags behind the reestablishment of pathogenic flora and propose a possibility of the clinical parameters returning to baseline, if there was a longer follow-up. Comparison of microbiological data obtained prior to and after therapy have supported the concept that there are distinct differences between healthy and diseased sites as therapy results in a reduction in the pathogenic motile rods and increase of the healthier coccoid forms. This response has been noted after root planing, antibiotic therapy, and surgical therapy.[24–26] However, studies have indicated that as bacterial flora repopulate the subgingival areas and return to baseline values, they are not necessarily accompanied by clinical changes. Despite this view, the presence of a pathogenic flora does indicate a need for subsequent monitoring of these sites and a more definitive eradication using antibiotics along with mechanical debridement. There was clinical improvement in groups A and B which correlated with an improvement in microbiological parameters, where a shift towards healthy flora was observed after 30 days and these results were maintained 90 days following therapy. The results of the present study are in agreement with those of earlier reports on local tetracycline used as an adjunct to mechanical debridement, where a significant change in the clinical and microbiological parameters was reported.[27]

The results of the present study have shown a significant reduction in the pathogenic flora and increase in healthy flora after using local metronidazole as an adjunct to mechanical debridement. These findings are in agreement with earlier studies where clinical and microbiological parameters were evaluated to assess the effect of local metronidazole therapy and significant changes were reported in all the parameters.[12,13] In the present study, we had put up multiplex PCR using primers for Aa, Pg, and Tf, as described in an earlier study.[29] An earlier study carried out by Garcia *et al.* described the procedure for identification of Aa, Pg, and *Prevotella intermedia* using multiplex PCR.[30] However, they did not identify Tf, which has been reported to be an important prognostic factor in longitudinal studies.[28] In the present study, although the number of samples was small, the prevalence of Pg and Tf was higher in sites prior to treatment in groups A and B. In group C, at the 90-days' posttherapy evaluation, Pg and Tf were still detected in higher numbers and this correlated positively with the microbiological findings. The study by Simon showed a presence of Aa, Pg, and Tf in sites with periodontitis.[29] We could detect Pg and Tf, but not Aa in our study, and as we did not use quantitative PCR, we could not quantify the changes seen before and after therapy. These two pathogens can be used as posttreatment markers, as described in a study by Osama Fujise *et al.* in 2002.[31]

Therefore, according to this study, there was no difference in the results achieved with local tetracycline or local metronidazole as adjuncts to mechanotherapy. However, both the antibiotic therapies resulted in greater improvement in microbiological parameters when compared to mechanotherapy alone.
